# Pathogenicity and virulence of *Phytophthora infestans*: The ever-evolving threat to food security and its sustainable management strategies

**DOI:** 10.1080/21505594.2025.2586882

**Published:** 2025-11-09

**Authors:** Wen-Jing Wang, Jun Li, Ge Zhao, Yan-Ping Wang, Shaobin Fan, Yu-Qing Dong, Li-Na Yang, Jiasui Zhan

**Affiliations:** a School of Agricultural Sciences, Zhengzhou University, Zhengzhou, China; b College of Chemistry and Life Sciences, Sichuan Provincial Key Laboratory for Development and Utilization of Characteristic Horticultural Biological Resources, Chengdu Normal University, Chengdu, China; c Key Laboratory on Conservation and Sustainable Utilization of Marine Biodiversity, Fuzhou Institute of Oceanography, Minjiang University, Fuzhou, China; d Department of Forest Mycology and Plant Pathology, Swedish University of Agricultural Sciences, Uppsala, Sweden; e College of Forestry, Sichuan Agricultural University, Chengdu, Sichuan, China

**Keywords:** Evolutionary ecology, effector proteins, host-pathogen interaction, sustainable agriculture, climate change, disease management

## Abstract

*Phytophthora infestans*, the oomycete pathogen responsible for late blight, remains a formidable threat to global potato and tomato production, causing significant economic losses and jeopardizing food security. This review synthesizes current knowledge of *P. infestans* and highlights its unique biology, sophisticated pathogenicity mechanisms, dynamic virulence factors, and management strategies. The pathogen employs a diverse arsenal of virulence factors such as effectors to suppress host immunity and manipulate cellular processes, while its genetic plasticity enables rapid adaptation to control measures. Environmental cues and host-pathogen co-evolution further complicate disease management, with climate change exacerbating these challenges. Despite advances in fungicides, resistant cultivars, and cultural practices, its ability to overcome control measures and evolve new virulence and functional traits underscores the need for innovative solutions. Emerging technologies including CRISPR-Cas9, RNA interference, and predictive modeling offer promising avenues for sustainable management of the pathogen. This review also calls for multidisciplinary approaches integrating genomics, ecology, and agronomy to develop durable strategies against *P. infestans* and ensure resilient agricultural systems in the face of evolving threats. Ultimately, this review provides a forward-looking perspective on how integrating these novel technologies ca with evolutionary-ecological principles can build sustainable and resilient management systems.

## Introduction

*Phytophthora infestans* is the causative agent of late blight, a devastating disease responsible for catastrophic losses in potato and tomato crops worldwide [1,2]. Unlike true fungi, *P. infestans* is characterized by its filamentous growth, water-dependent spores, and host-specific pathogenicity [3,4]. The pathogen thrives in cool, humid environments, and rapidly invades plant tissues, leading to necrotic lesions, wilting, and crop collapse within days under favorable conditions [5,6].

The historical significance of *P. infestans* is inextricably linked to the Irish Potato Famine (1845–1852), which triggered widespread starvation, mass migration, and socio-economic upheaval [3,7]. Today, late blight remains a persistent threat to global food security, causing annual economic losses exceeding 6 billion USD due to reduced yields, expensive fungicide applications, and post-harvest spoilage [8]. Modern agricultural systems face renewed challenges from emerging *P. infestans* strains that exhibit increased virulence, fungicide resistance, and adaptability to changing climates [9].

Studying the pathogenicity and virulence mechanisms of *P. infestans* is critical for developing sustainable disease management strategies. The abilities of the pathogen to secrete effector proteins, evade host immune responses, and rapidly evolve new virulence traits highlight the complexity of host-pathogen interactions [10,11]. Advances in genomics, molecular biology, plant breeding, and computational biology have shed light on the dynamic evolution of *P. infestans* [12], yet gaps persist in understanding how environmental factors and genetic diversity drive its destructive potential [13]. Here we synthesize current knowledge on the biology, virulence determinants, and interactions of the pathogen with host plants and environments, while highlighting innovative approaches to mitigate its impact on agriculture. By bridging these fundamental research and practical applications, this review aims to guide future efforts against this formidable pathogen. We first establish a foundation of its biology and molecular weaponry, then explore the evolutionary dynamics of its threat, and finally synthesize integrated management strategies. A key contribution is our critical evaluation of how emerging technologies, applied within an evolutionary framework, can achieve sustainable control.

## Taxonomy and classification

*P. infestans* is a member of the kingdom *Chromista* and the phylum Oomycota, a group of filamentous, eukaryotic microorganisms that have historically been misclassified as fungi due to their superficial similarities in growth and ecology [14]. However, oomycetes are phylogenetically distinct, belonging to the *Stramenopiles* lineage [15], which includes diatoms, brown algae, and water molds. *Stramenopiles* are characterized by heterokont flagellation, defined by motile zoospores possessing two morphologically distinct flagella: a forward- pointing, hairy flagellum used for propulsion and a trailing, straight and hairless flagellum used for steering [16]. Unlike true fungi, oomycetes lack chitin in their cell walls, which are instead composed of cellulose and *β*-glucans, and their life cycles are primarily diploid, in contrast to the dominant haploid cycles of fungi [17].

Within the taxonomic hierarchy of the kingdom, *P. infestans* is further classified under the class *Oomycetes*, order *Peronosporales*, and family *Peronosporaceae* [18]. This group is often referred to as the “downy mildews” due to their shared features with other members such as *Plasmopara viticola*. The genus *Phytophthora* comprises over 220 species [19] and many of them are notorious plant pathogens. *P. infestans* resides in clade 1c of the *Phytophthora* phylogenetic tree [20], a subgroup that primarily includes pathogens targeting *Solanaceous* crops such as potatoes and tomatoes. The species is heterothallic, requiring two complementary mating types for sexual reproduction. This feature promotes genetic diversity and adaptability by generating recombinant offspring with novel virulence traits. However, self-fertile strains have been detected in several countries including China [21,22], the leading potato production region in the world [23].

The placement of *P. infestans* in *Phytophthora* Clade 1c offers a practical framework for disease management. While shared biology within the clade such as conserved RXLR and CRN effector repertoires (Table 1) means pathogenicity insights can be extrapolated between species, critical ecological differences dictate separate strategies. The aerial, foliar blight caused by *P. infestans* requires foliar fungicide applications, in stark contrast to the soil-focused management needed for the root rots caused by clade-mates like *P. sojae*. Consequently, this phylogenetic knowledge is essential for predicting control measure efficacy, guiding chemical screening, and deploying clade-specific resistance genes from wild relatives.

**Table 1.** Comparative biology and disease management implications of *Phytophthora infestans* and selected Phytophthora species from different phylogenetic clades. The comparison with *P. sojae* (clade 1b) highlights shared evolutionary strategies within a major lineage, while with *P. cinnamomi* (clade 8) illustrates divergent adaptations in a broad-host-range pathogen.

Feature	*P. infestans* (Clade 1c)	*P. sojae* (Clade 1b)	*P. cinnamomi* (Clade 8)
Primary Hosts	Potato, Tomato (*Solanaceae*)	Soybean	> 5000 species (e.g. avocado, oak)
Infection Site	Aerial (leaves, stems)	Roots & stems	Roots & lower stem (collar rot)
Key Dispersal Unit	Airborne sporangia	Soil/water-borne zoospores	Soil/water-borne zoospores
Dominant Reproductive Strategy in Epidemics	Asexual (clonal lineages)	Asexual (clonal lineages)	Asexual (clonal lineages)
Representative Effector Arsenal	Large, diverse RXLR & CRN families	Large, diverse RXLR & CRN families	Distinct effector repertoire; fewer canonical RXLRs
Practical Management Consequences	Foliar fungicides, canopy management, aerial resistance genes	Seed treatments, soil amendments, root resistance genes	Soil fumigation, host avoidance, water management
Source of Key Resistance (R) Genes	Wild *Solanum* spp. (e.g. *S. demissum*, *S. bulbocastanum*)	Wild *Glycine* spp.	Limited; resistance is often polygenic and quantitative

## Life cycle and infection process

**Figure 1.**
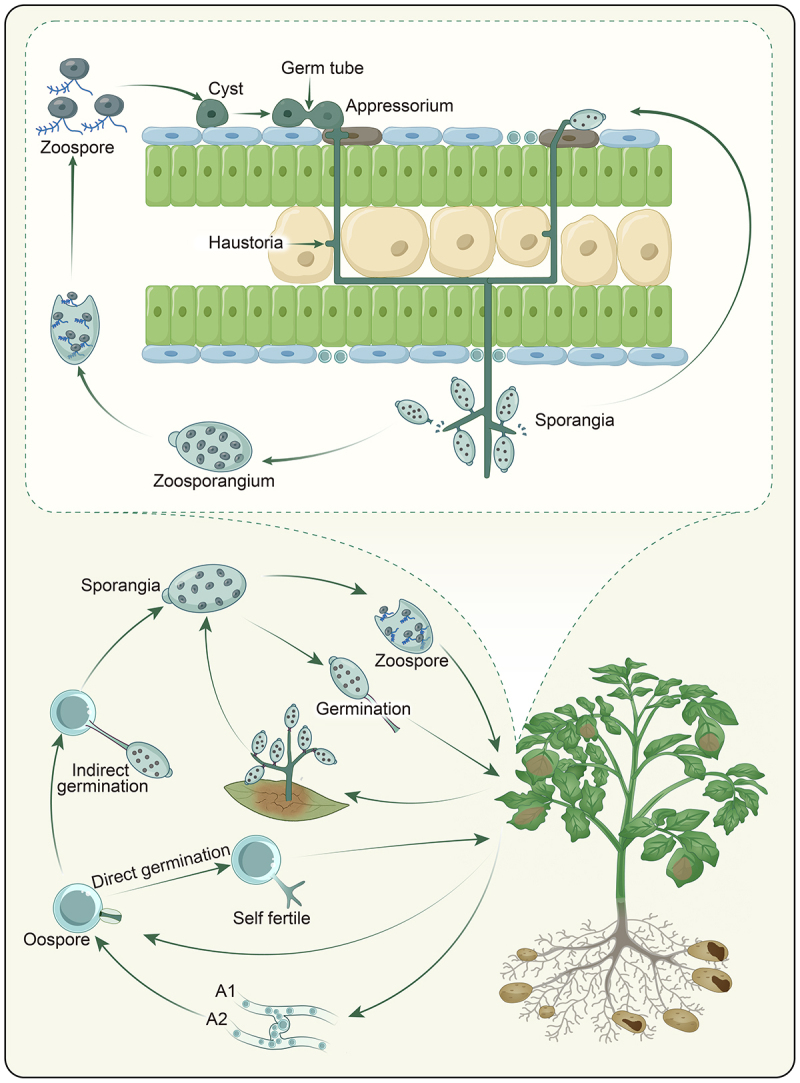
In the asexual cycle, sporangia produced on sporangiophores either germinate directly under warm conditions or release motile zoospores in cooler, wet environments. Zoospores encyst and form penetration hyphae that invade host tissues through stomata or wounds. The sexual cycle involves outcrossing between compatible mating types or selfing, producing thick-walled oospores that persist in soil for years and serve as reservoirs for new infections. These oospores can germinate directly or indirectly to initiate disease. The infection cycle begins when sporangia or cysts germinate into germ tubes, which typically form appressoria to penetrate epidermal cell walls (though stomatal entry may also occur). Intercellular hyphae subsequently colonize the leaf tissue, and sporangiophores emerge through stomata 3–5 days post-infection to produce new sporangia, completing a single infection cycle. Repeated cycles drive late blight epidemics.

Both asexual and sexual reproductive strategies in the life cycle (Figure 1) of *P. infestans* enable its epidemiological and evolutionary success [24]. The asexual cycle dominates during active epidemics, characterized by the production of sporangia on branched sporangiophores that emerge from infected tissues. These sporangia can either germinate directly under warm conditions or release biflagellate zoospores at lower temperature, which serve as the primary dispersal units [24]. In contrast, sexual reproduction occurs when compatible mating types interact or in self-fertile strains, leading to the formation of oospores [25]. These thick-walled oospores represent a survival strategy, persisting in soil for at least 2–3 years and providing genetic recombination that generates novel pathogenic strains [26]. The relative importance of these reproductive modes varies geographically, with sexual reproduction becoming increasingly significant in regions where both mating types coexist such as in South America [27] and Scandinavia [28,29].

The infection process initiates when sporangia land on susceptible plant surfaces. Under optimal conditions, germination of a sporangia or cyst formed by zoospores on the leaf surface produces a germ tube [30]. Assisted by secreted cell wall-degrading enzymes including pectate lyases and cellulases, the germtube then forms specialized infection structures called appressoria that generate mechanical pressure to physically breaking the cuticle [31,32]. Penetration can also occur through stomata in leaves [33] while tuber infections typically initiate through lenticels or wounds [34].

Following successful penetration, *P. infestans* establishes an initial biotrophic phase in which haustoria forms intimate contact with living host cells [35]. These finger-like hyphal extensions invaginate the host cell membrane without breaking it, creating a specialized interface for nutrient uptake and effector delivery. About 72–96 hours after infection, the pathogen switches to necrotrophy, marked by extensive branching of hyphae through intercellular spaces and subsequent cell death [36]. During the infection phase, the pathogen secretes a cocktail of apoplastic and cytoplasmic effectors that suppress plant immunity and reprograms host metabolism.

The reproductive phase begins 3–5 days after the initial infection when sporangiophores emerge through stomata [33]. *P. infestans* can forcibly open stomata by releasing specific pathogenicity factors such as effector proteins [37]. This phase is often visible as white fuzzy growth on lesion margins [38]. Each sporangiophore can produce 5–10 sporangia in a single day, with each sporangium capable of initiating new infections [39]. Sporangial detachment and dispersal occur primarily through rain splash or wind, with dispersal distances ranging from a few centimeters to several kilometers under storm conditions. This secondary spread creates characteristic “foci” of infection in fields [40,41].

Disease development exhibits strict environmental dependence, with temperature and moisture being the primary determinants [41]. Optimal temperatures for mycelial growth and sporangial production are geographically dependent [42], ranging between 15°C and 20°C while zoospore release requires 10–15°C. Critical to infection is the duration of leaf wetness, with a minimum of 8–12 hours required at optimal temperatures [43]. Relative humidity > 90% sustains sporulation while values < 80% inhibit sporangial formation [6]. Under favorable climatic conditions, the levels of the disease can grow exponentially as the pathogen can complete a generation in 3–4 days. Therefore, these climatic parameters form the basis of disease forecasting systems [44,45]. Regarding future epidemics, climate change models predict altered disease patterns, with warmer temperatures potentially reducing epidemic risk in some regions while increasing it in others through changes in dew formation patterns and rainfall distribution [46,47].

The development of late blight is critically dependent on environmental conditions, primarily determined by temperature and leaf wetness. As detailed in Table 2, each critical phase of the pathogen’s life cycle relies on a distinct set of optimal conditions. These precise requirements form the scientific basis for modern forecasting systems like Blitecast and Plant-Plus, which integrate real-time weather data to predict infection risk and optimize fungicide timing. Understanding these parameters is therefore essential both for understanding the biology of *P. infestans* and for implementing effective, timely control strategies in the field.

**Table 2.** The specific environmental requirements for each critical phase in the life cycle of *Phytophthora infestans*. The development of the pathogen is strictly dependent on precise combinations of temperature and moisture, creating predictable windows of infection risk. The parameters listed here form the scientific foundation for modern disease forecasting systems.

Phase/Process	Optimal Temperature (°C)	Critical Moisture/Humidity Requirement	Practical Significance & Management Insight
**Sporangial Germination** (Direct)	15 – 20	Leaf wetness (8 – 12 hours minimum)	This defines the primary infection window. Disease forecasting models use these parameters to issue “infection warnings,” signaling the need for protective fungicide application.
**Zoospore Release** (Indirect)	10 – 15	Free water for sporangial cleavage	Important in cooler, water-saturated conditions. Drives epidemics in areas with prolonged rain or dew.
**Penetration & Infection**	15 – 20	Sustained leaf wetness during germination	The success of this phase justifies the use of protectant fungicides that form a barrier on the leaf surface before the infection period begins.
**Sporulation** (Sporangiophore production)	15 – 20	Relative Humidity > 90%	High humidity enables massive production of inoculum for secondary spread. Canopy management to reduce humidity is a key cultural control.
**Sporangial Survival & Dispersal**	Cool (10–15°C) prolongs survival	Low humidity ( < 60%) leads to rapid desiccation	Sporangia can be wind-dispersed over long distances under dry air conditions, leading to new disease foci. Explains the rapid spread across regions.
**Oospore Germination**	10 – 15	Saturated soil moisture	The germination of this sexual overwintering structure depends on specific conditions to initiate primary infections, making crop rotation a critical strategy for reducing soil inoculum.

## Molecular mechanisms of pathogenicity

*P. infestans* has evolved a sophisticated and highly adaptable virulence strategy that integrates molecular, biochemical, and ecological mechanisms to ensure successful host colonization and rapid adaptation [42]. Central to its success is an extensive effector repertoire, including RXLR (Arg-X-Leu-Arg) and CRN (Crinkling and Necrosis) proteins, which enable the pathogen to manipulate host defenses with remarkable precision [43]. Beyond effector-mediated virulence, *P. infestans* employs a coordinated enzymatic assault to disrupt and macerate host tissues, simultaneously suppressing host defense systems and facilitating nutrient acquisition [44].

### Effector proteins

The effector repertoire of *P. infestans* represents one of the most sophisticated virulence systems among plant pathogens, enabling it to overcome host defenses and infect potato and tomato crops [35,48]. These effectors fall into two functional classes: (1) apoplastic effectors such as protease inhibitors and lectins that neutralize antimicrobial compounds and inhibit pathogenesis-related proteins in the extracellular matrix to create favorable infection conditions; and (2) cytoplasmic effectors including RXLR and CRN types that disrupt intracellular immune signaling. In addition to immunosuppression, effectors actively remodel host physiology by degrading cell walls [49,50], altering membrane transport [51,52] and hijacking metabolism to redirect nutrients toward pathogen growth [53–55]. This adaptable effector arsenal highlights the evolutionary success of *P. infestans* as a destructive plant pathogen.

The RXLR effector family, named for its conserved N-terminal Arg-X-Leu-Arg motif that facilitates host cell translocation, is a cornerstone of *P. infestans* pathogenicity, exhibiting extraordinary structural and functional versatility. While this motif is essential, recent studies indicate that additional C-terminal motifs also contribute to host cell entry [48,56]. Structural biology approaches have revealed that RXLR effectors often mimic eukaryotic protein folds, enabling them to interface with host immune components with remarkable specificity [56,57]. Experimental evidence from model effectors provides this foundation; for instance, the solved structure of Avr3a revealed a modular architecture essential for host target specificity [57,58].

RXLR effectors employ a multi-pronged approach to disrupt plant immunity at various levels. Early in infection, effectors prevent pathogen recognition by physically blocking the binding sites of immune receptors or promoting their degradation [58]. Some effectors such as Avr1 and Avr3 target the plant vesicle trafficking system, interfering with the delivery of defense-related compounds to infection sites [59]. The effector PexRD54 exploits host autophagy by competitively binding ATG8 proteins, thereby repurposing this cellular clearance system for pathogen benefit [53–55]. Recent work has also identified RXLR effectors that modify host chromatin structure or small RNA pathways, representing an additional layer of immune interference [60,61].

The expression of RXLR effectors is tightly regulated in a temporal cascade during infection, with distinct waves of effectors targeting successive layers of plant immunity [62]. This phased deployment mirrors the guard hypothesis [36,63] in which early expressed effectors initially suppress PAMP-triggered immunity (PTI), followed by later effectors that counteract effector-triggered immunity (ETI). This sophisticated temporal strategy resembles a molecular version of Wolfe’s “trench warfare” model of host-pathogen interaction [64], in which each wave of effectors probes and subverts the defense responses of plants. The coordinated action suggests an exquisite evolutionary adaptation that allows *P. infestans* to dynamically adjust its virulence strategy throughout the infection process.

CRN effectors represent another major class of virulence proteins that enable *P. infestans* to systematically dismantle plant defenses and reprogram host cells. This pathogen encodes 196 CRN effectors alongside 255 pseudogenes, forming its second-largest effector family after RXLRs [35]. This represents significantly expanded CRN repertoires relative to related oomycetes, for example,100 genes/102 pseudogenes in *P. sojae* and 19 genes/42 pseudogenes in *P. ramorum* [65]. This expansion parallels RXLR effector genes and is attributed to their location in repeat-rich, gene-sparse genomic regions enriched with transposable elements. Structurally, CRNs resemble RXLR effectors as modular secreted proteins with conserved N-terminal motifs (LXLFLAK, DWL, and HVLVXXP) essential for host translocation, and highly diverse C-terminal domains responsible for effector functions [65,66]. However, CRNs exhibit greater sequence conservation than RXLRs [65]. These effectors exploit structural mimicry to deregulate phosphorylation-based signaling, hijack transcriptional machinery in the nucleus, and disrupt critical processes like gene expression and organelle function, making them central to pathogenicity [35,65].

CRN effectors are predominantly highly expressed during infection. Approximately 50% of CRN-encoding genes rank among the top 1% of most highly expressed genes in *P. infestans* [67]. This expression exhibits both organ specificity and temporal regulation, varying across infection stages and host tissues [65,68]. The high expression levels underscore their critical role in establishing and maintaining infection, aligning with their functions in manipulating host immunity and physiology at specific developmental phases of the pathogen [69]. In stark contrast to the well-characterized RXLR effectors, the functions of CRN effectors in *P. infestans* remain largely unknown. Of the 196 CRNs it encodes, only three (CRN1, CRN2, and CRN8) have been partially characterized. The presumed roles of the vast majority are hypothetical, inferred from their high expression during infection, their modular structure, and studies of their homologs in related *Phytophthora* species.

Despite their abundance and high expression, functional characterization of CRN effectors in *P. infestans* lags significantly behind RXLR effectors. Only three members (CRN1, CRN2, and CRN8) have been extensively studied. CRN1 and CRN2 were initially identified via functional expression screening in plants using a Potato virus X vector. Their expression in *Nicotiana* spp. and tomato induces leaf crinkling, cell death, and defense gene induction [67]. Deletion analysis of CRN2 identified a minimal 234-amino acid C-terminal region (DXZ domain, aa 173–407) sufficient for cell death induction, with other C-terminal domains (DC, DBF, D2, DXW-DXX-DXS) also triggering cell death [35,66]. CRN8 requires nuclear accumulation to induce host cell death and possesses a predicted RD kinase domain that targets host factors to perturb defenses [35,65]. It plays a critical stage-specific role by activating programmed cell death during the necrotrophic phase [65,70]. While CRNs are proposed to be cytoplasmic effectors based on modularity akin to RXLRs and translocation has been observed when a putative CRN translocation motif is fused to Avr3a C-terminal domain, direct experimental evidence demonstrating CRN N-terminus-mediated translocation into plant cells remains unreported [65]. The vast majority of the 196 CRNs remain functionally uncharacterized, representing a significant knowledge gap in *P. infestans* pathogenesis. Research from other species shows nuclear-targeted CRNs epigenetically reprogram host defenses through diverse mechanisms: some induce DNA hypermethylation at defense gene promoters while promoting demethylation at sugar transporter genes, simultaneously silencing immunity, and enhancing nutrient flux [35,65]. Others mimic plant transcription factors, competitively binding cis-regulatory elements (e.g. G-boxes on JA-responsive gene promoters) to block defense gene activation. Certain nuclear CRNs even exhibit topoisomerase I-like activity or induce DNA damage [71]. Chloroplast-targeted CRNs cripple photosynthetic efficiency and redirect carbon resources. CRNs also display dual, stage-specific functions [69,72].

### Enzymatic attack

The enzymatic attack by *P. infestans* represents a precisely coordinated campaign against plant structural defenses. The pathogen secretes a carefully balanced cocktail of cell wall-degrading enzymes (CWDEs) including pectinases, cellulases, and hemicellulases [73,74]. The production of these enzymes is tightly regulated both temporally and spatially during infection, allowing the pathogen to penetrate plant surfaces while minimizing the release of immunogenic oligosaccharides.

Pectinases including polygalacturonates and pectin lyases are among the first enzymes deployed to soften the middle lamella between plant cells. These are followed by cellulases and hemicelluloses that target the structural framework of the cell wall [73,75]. The pathogen avoids triggering excessive damage responses by producing these enzymes in controlled amounts and often in truncated or modified forms that evade plant immune recognition [76,77]. Recent proteomic studies have identified several novel CWDEs with unusual substrate specificities, including enzymes that target callose and other defense-related cell wall reinforcements [78,79]. Some of these enzymes work synergistically with effectors. For example, certain pectinases create oligogalacturonide fragments that are then bound by pathogen proteins to prevent their recognition as damage-associated molecular patterns [76]. CWDE families exhibit host-specific expansions, with potato-adapted strains encoding more pectinase gene copies than those infecting tomatoes, highlighting the role of enzymatic profiling in host adaptation [73,80].

### Defense suppression

Manipulation of plant defense systems by *P. infestans* extends far beyond simple suppression. Instead, it represents a comprehensive reprogramming of host physiology. Hormonal manipulation is particularly sophisticated, with the pathogen able to both synthesize hormone mimics and interfere with endogenous hormone signaling pathways [81,82]. SA signaling is suppressed through multiple mechanisms. Some effectors directly target the SA biosynthesis pathway while others promote SA degradation or interfere with NPR1, the central regulator of SA responses [83,84]. Simultaneously, the pathogen activates JA and ethylene signaling in a carefully balanced manner that prevents effective defense activation while avoiding excessive cell death that may be detrimental to its biotrophic growth [85,86]. *P. infestans* also manipulates abscisic acid signaling to regulate stomatal closure and interferes with strigolactone pathways to alter plant architecture [82,83]. This hormonal manipulation is precisely timed, with different effectors targeting distinct pathways at specific infection stages, creating a “defense confusion” strategy that renders the host unable to mount an effective response.

### Nutrient acquisition

Nutrient acquisition is another critical component of virulence. The haustoria of *P. infestans* represent highly specialized and dynamic interfaces for nutrient acquisition and molecular communication. These structures undergo continuous remodeling during infection, with their morphology and function changing as the infection progresses [87,88]. Early in infection, haustoria are small and fragile, optimized for stealthy nutrient uptake. Whereas in late phase of the infection, they become more robust to support massive nutrient flows. Advanced imaging techniques have revealed that haustoria establish an extensive membrane network that increases the surface area for transport while minimizing direct cytoplasmic contact.

The pathogen expresses a specific set of nutrient transporters in haustoria, including hexose transporters optimized for plant sugars and amino acid transporters with particular affinity for glutamine and asparagine. During the necrotrophic phase, the pathogen shifts to more aggressive nutrient acquisition by secreting a broader range of degradative enzymes and activating additional transporter systems to capture the breakdown products of dying host cells [87,88]. This two-phase strategy allows the pathogen to maximize resource extraction while minimizing early detection by host surveillance systems.

## Evolutionary adaptation of P. INFESTANS

The evolutionary adaptability of *P. infestans* is unparalleled among plant pathogens, enabling it to overcome host defenses, evade chemical controls, and thrive in diverse environments. This adaptability stems from the interconnected set of biological and genomic features that together drive the formation of genetic diversity, effector repertoire plasticity, environmental responsiveness and host specialization, and allow the pathogen to swiftly overcome new host resistance genes through effector evolution, adapt to changing environmental conditions through selective sweeps, and develop fungicide resistance through targeted mutations.

### Genetic diversity

The remarkable evolutionary plasticity of *P. infestans* arises from multiple synergistic mechanisms that drive the generation of genetic variation in a population-level. Sexual recombination, occurring both through outcrossing when compatible A1 and A2 mating types coexist and via self-mating in homothallic strains [89,90], plays a pivotal role. This process generates novel genotypic combinations through meiotic chromosome reshuffling, with particularly significant impacts in two key regions: Mexico, the putative center of origin [91] where both mating types naturally coexist [92] and Scandinavia, where harsh winters select for the durable oospores produced through sexual reproduction [93]. In contrast to regions with active sexual recombination, most major potato-growing areas are dominated by clonal lineages, such as the historic US-1 and the more recent US-8 and US-23. This clonal population structure dictates local evolutionary dynamics and outbreak patterns. Consequently, monitoring the spread and prevalence of these dominant lineages is a critical management activity. Tracking key traits including fungicide sensitivity, virulence spectra, and aggressiveness provides essential data to guide control strategies, such as selecting effective fungicides and deploying cultivars with corresponding R genes.

The pathogen contains numerous active transposable elements [35,75]. This genome architecture further enhances its variability by inducing mutations and large-scale chromosomal rearrangements. First identified through comparative genomics of *P. infestans, P. sojae*, and *P. ramorum*, this “two-speed” genome structure – with effector genes localized in repeat-rich, gene-sparse regions is now a recognized hallmark of the *Phytophthora* genus. Comparative genomic analyses have revealed extensive structural variations within and among geographic populations, including gene duplications that create functional redundancy [94,95], targeted deletions of genes associated with critical biochemical pathways [85,96], and chromosomal inversions that alter gene expression profiles [97,98]. Heterokaryosis has also been observed [99,100], enabling additional avenues of the pathogen to generate genetic variation through somatic recombination.

### Effector repertoire variability

The effector repertoire of *P. infestans* exhibits remarkable evolutionary dynamics that enable the pathogen to continuously adapt to host defenses. At the molecular level, effector genes undergo rapid evolution through multiple mechanisms. Effector families show strong signatures of positive selection [75,101], with elevated nonsynonymous mutation rates in functional domains that allow escape from host recognition while maintaining virulence functions [102]. Additional genetic modifications, including point mutations, altered start/stop codons, and changes to protein disorder regions [103,104], further contribute to effector diversification. The pathogen also employs epigenetic silencing or alternative splicing to generate phenotypic variation from conserved genetic templates [105,106], providing another layer of adaptability.

The genomic architecture of *P. infestans* strongly facilitates effector evolution. Effector genes are strategically located in gene-sparse, repeat-rich regions of the genome that are highly prone to recombination and structural variation [35,75]. This genomic architecture is conserved across the genus. For example, while the RXLR effector superfamily is massively expanded in both *P. infestans* and *P. sojae* and shows strong signatures of positive selection, reflecting a shared arms race with their hosts, the CRN effector family shows lineage-specific variation (196 genes in *P. infestans*, ~100 in *P. sojae*, and 19 in *P. ramorum*). This demonstrates that while the repeat-rich genomic hotspots driving effector evolution are a genus-wide feature, the specific expansion of individual effector families represents a lineage-specific adaptation to host ecology. These genomic “hotspots” experience frequent gene duplication events, creating paralogous effector sets that provide functional redundancy while allowing for specialization. The presence of numerous transposable elements in these regions promotes additional genetic rearrangements and horizontal gene transfer events of effectors, further expanding the toolkit. At the population level, this effector plasticity is further enhanced by the pathogen’s mating system. Inter- and intra-gene recombination facilitates the generation of novel effector alleles and/or combinations [101,107], while a heterokaryotic strategy enables somatic recombination of effector loci [100,101].

### Environmental adaptability

*P. infestans* demonstrates extraordinary phenotypic plasticity in response to environmental cues. Temperature adaptation range of the pathogen is maintained through differential expression of heat-shock functional genomes [103] but it has been hypothesized that it can be quickly adjusted according to real time thermal conditions [108,109]. Transcriptomic studies in other species of *Phytophthora* also reveal distinct gene expression profiles under varying moisture conditions, with upregulation of osmoregulation genes during drought stress and activation of motility genes in water films [110,111]. The pathogen can also modulate its life cycle strategy based on environmental conditions, favoring rapid asexual reproduction during optimal conditions but switching to sexual reproduction for genetic diversity and survival under stresses [29]. This adaptability explains its successful establishment across diverse agro-ecological systems from cool highland regions to warmer lowland areas.

### Host specificity

Host specialization has been documented at multiple biological levels, from broad host range differences to cultivar-specific interactions. Genome comparisons reveal lineage-specific gene expansions, particularly in effector families, that correlate with host preference. Comparative analysis reveals that host-specific CAZyme adaptation is a general pattern across *Phytophthora* species. For instance, potato-adapted *P. infestans* strains exhibit expansions in pectinases tailored to *Solanaceous* cell walls, whereas the soybean pathogen *P. sojae* possesses a distinct CAZyme profile optimized for its host. This highlights how lineage-specific enzymatic profiling is a key driver of host adaptation within the genus. At the molecular level, host specificity is mediated by allelic variation in effector proteins that determine compatibility with host immune receptors and even ecological conditions [112]. Population genomic studies have identified distinct subpopulations specializing on wild *Solanum* species versus domesticated potatoes [113] with different virulence patterns. The ability to rapidly overcome host resistance is not unique to *P. infestans*. The soybean pathogen *P. sojae* routinely adapts to major Rps genes through mutations in corresponding Avr effector genes, demonstrating that effector-driven adaptation is a fundamental, genus-wide evolutionary strategy. The pathogen can rapidly adapt to new hosts through epigenetic modifications that alter effector expression profiles [114], providing a mechanism for host range expansion without genetic changes. This specialization creates complex pathosystems in which virulence must be considered relative to specific host genotypes and environments.

## Disease management strategies

**Figure 2.**
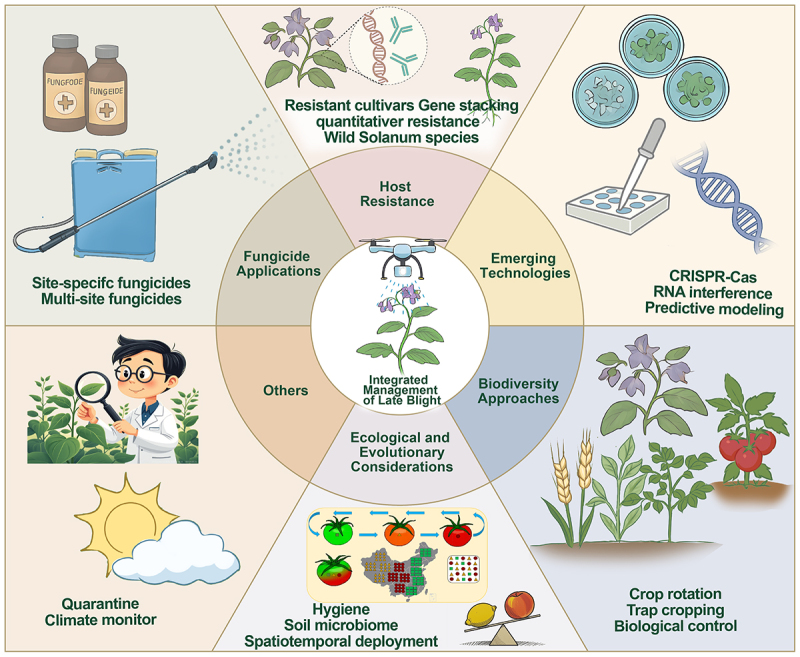
Integrated management strategy for *Phytophthora infestans*-induced late blight in potato and tomato crops. Key components include: 1) resistant cultivars developed via traditional breeding or genetic engineering (e.g. stacking R genes or quantitative resistance traits), incorporating genetic resources from wild Solanum species for enhanced durability; 2) emerging technologies, such as CRISPR-Cas9 for editing host susceptibility genes, rna interference (RNAi) for targeting essential pathogen genes, and predictive modeling with IoT-based monitoring for early detection and intervention; 3) biodiversity approaches, including crop rotation with non-host species (e.g. cereals, brassicas) to disrupt pathogen life cycles, trap cropping to divert pathogen pressure, and application of antagonistic microorganisms as biological controls;4) ecological and evolutionary considerations, such as removal of alternative hosts to reduce pathogen reservoirs and implementation of climate-adaptive strategies; 5) strategic fungicide use, including site-specific and multi-site fungicides to curb resistance evolution, supported by precision technologies like electrostatic sprayers and UAV-based systems to minimize environmental impact; 6) other management measures, including enhanced disease quarantine and climate change surveillance.

Effective management of late blight requires an integrated approach combining multiple strategies tailored to local conditions. Rapid evolution and adaptability of the pathogen necessitate comprehensive solutions addressing both immediate control and long-term sustainability. Successful programs integrate host resistance, chemical control, biodiversity conservation, and innovative technologies (Figure 2) while accounting for the ongoing co-evolutionary arms race between pathogen and host. Modern management systems must balance efficacy with environmental impact, economic feasibility, and social acceptability across diverse agricultural contexts from smallholder farms to large-scale commercial operations.

### Host resistance

Modern late blight management relies heavily on developing resistant potato cultivars through two complementary genetic approaches. Qualitative resistance mediated by single dominant R genes provides complete but often short-lived protection by recognizing specific pathogen effectors and triggering hypersensitive cell death responses. Over 20 R genes have been identified from wild *Solanum* species [115 ,116]. However, rapid effector evolution overcomes these R genes through mutations in targeted Avr proteins within 3–7 growing seasons.

Quantitative resistance offers more durable protection through the synergetic function of multiple genes to influence various defense mechanisms. These include enhanced cell wall lignification, increased production of antimicrobial compounds, and improved stomatal regulation to limit infection [71,117,118]. Varieties like Sarpo Mira demonstrate how combining multiple partial resistance traits can maintain field resistance for a decade or more [119]. However, quantitative resistance does not completely prevent disease and therefore must be used in conjunction with other control measures to ensure successful production. In addition, quantitative resistance may select for greater pathogenicity and tolerance to ecological stresses [,^116^], potentially jeopardizing long-term sustainable production.

Current breeding programs increasingly focus on pyramiding approaches that combine the best features of both resistance types. By stacking multiple R genes with quantitative traits, breeders aim to create cultivars with both strong initial protection and long-term durability. Modern techniques like marker-assisted selection and genomic prediction accelerate this process by enabling precise introgression of resistance in wild species while minimizing linkage drag. Emerging cisgenic approaches, which use only native potato DNA, may improve public acceptance of resistant varieties while maintaining genetic diversity [120,121].

Supported by improved understanding of resistance gene networks, the most advanced resistant cultivars now incorporate 3–5 R genes along with multiple quantitative trait loci [122,123]. However, continued pathogen evolution requires constant identification of new resistance sources including novel NLR genes from *S. americanum* and *S. verrucosum* [124]. Furthermore, due to the rapid evolution of *P. infestans*, stacking multiple resistance genes into a single variety may lead to the simultaneous loss of resistance genes. To justify the breeding philosophy for the preservation of resistant resources, it is necessary to evaluate the average persistence of resistance in both pyramiding and conventional strategies.

### Fungicide applications

Chemical management of late blight remains essential for commercial production of potato and has undergone significant evolution in recent years, driven by technological advances and the need to combat pathogen resistance. Modern fungicide programs combine different chemical classes with precision application technologies to maximize efficacy while minimizing environmental impact. Site-specific fungicides such as quinone outside inhibitors and carboxylic acid amides provide targeted action against key pathogen processes [125,126]. However, their specificity makes them highly vulnerable to resistance, as demonstrated by the emergence of mandipropamid (a CAA fungicide) resistant genotypes of *P. infestans* in Europe [127]. They are typically alternated with multi-site fungicides such as copper compounds and chlorothalonil or mancozeb which are less prone to resistance development and play a crucial role in resistance management strategies [128,129]. Application frequencies have become more risk-based, ranging from < 7-day intervals during high disease pressure periods to 14-day schedules when conditions are less favorable for disease development. Recent studies emphasize the importance of mixing different modes of action within single applications to delay resistance evolution.

The new generation chemistry has brought innovative solutions to late blight control. Oxysterol binding protein inhibitors like oxathiapiprolin demonstrate remarkable efficacy by disrupting lipid metabolism in the pathogen [130–132]. Other novel compounds including succinate dehydrogenase inhibitors target essential energy production pathways. These next-generation fungicides often have unique modes of action that make them effective against strains resistant to conventional chemistries [133]. However, their high specificity also makes resistance management protocols particularly critical and manufacturers now routinely recommend strict rotation schedules and combination treatments from outset of product launch.

Application technology advancements show promise for improving fungicide delivery systems. For instance, electrostatic sprayers can potentially achieve improved leaf surface coverage through charged droplet technology [134,135], though their efficacy in penetrating dense potato canopies requires further validation. Similarly, Unmanned Aerial Vehicle (UAV)-based systems are being explored for precise, low-volume applications in agriculture [136,137]. However, it is important to note that methods such as electrostatic sprayers demonstrate concepts like spot-treatment for weeds or applications in less dense crops; their operational feasibility and consistent efficacy for managing late blight in dense potato foliage are still under development and face challenges such as ensuring adequate canopy penetration and deposition uniformity. Modern decision support systems such as Plant-Plus integrate real-time weather data, disease risk models and crop growth stages to optimize application timing. These technological improvements not only enhance control efficacy but also support sustainability goals by minimizing chemical inputs, reducing operator exposure, and mitigating environmental contamination.

### Ecological approaches

Ecological strategies offer sustainable alternatives for late blight management by harnessing natural systems and processes. These approaches work in harmony with conventional methods to create more resilient agricultural ecosystems. Cultural practices form the foundation of ecological disease management. Implementing 3-year rotations with non-host crops like cereals and brassicas reduced soil inoculum substantially [138,139]. These rotations disrupt the life cycle of the pathogens while improving soil health. Furthermore, managing primary inoculum sources such as volunteer potato plants, wild *Solanum* weeds, and potato cull piles through systematic sanitation can significantly reduce initial infection pressure in fields. The general principle that crop diversification suppresses disease suggests that intra-potato diversification may substantially reduce late blight severity [140,141]. By analogy to other pathosystems, this approach could also slow the evolution of *P. infestans* pathogenicity and fungicide resistance, and enhance beneficial microbial communities [141,142]. Biofumigation techniques employing *Brassica juncea* release glucosinolates that suppress soilborne inoculum through natural fungicidal activity. When incorporated at flowering stage, these green manures can substantially reduce oospore viability [143,144]. Complementary practices like delayed planting to avoid peak disease periods and optimized irrigation timing further enhance these effects.

Biological control is gaining traction as viable components of integrated management. *Bacillus subtilis* QST713 has demonstrated consistent disease reduction in field trials through multiple mechanisms including antibiotic production and induced systemic resistance [145]. *Pseudomonas fluorescens* strains producing phenazine-1-carboxylic acid show particular promise for tuber protection, reducing late blight incidence in storage [146]. *Mycoparasitic* fungi like *Trichoderma atroviride* actively attacks *P. infestans* hyphae and spores while stimulating plant defense responses [147]. Emerging research is exploring consortia of beneficial microorganisms that work synergistically, with some combinations achieving control levels comparable to chemical fungicides in low-pressure situations.

These ecologically based approaches are most effective when combined with customized protocols for specific sites. While generally requiring more management knowledge than conventional methods, they offer long-term sustainability benefits including reduced risk of fungicide resistance, improved soil health, and decreased negative impacts on environments.

### Emerging technologies

Innovations in cutting-edge technology are transforming control efforts of plant diseases and will revolutionize the way late blight is managed. These advanced solutions offer more targeted, sustainable, and precise approaches to disease control while addressing the remarkable adaptability of the pathogen.

Genetic technologies are pushing the boundaries of crop protection. CRISPR-based editing of SWEET sugar transporters in potato plants has shown significant reduction in late blight severity by starving the pathogen of essential carbohydrates [148,149]. Host-induced gene silencing systems now target multiple essential pathogen genes simultaneously, including cellulose synthase and effector genes, achieving remarkable protection in field trials [150,151]. Synthetic biology approaches are engineering beneficial microbiome communities that not only suppress *P. infestans* through competition but also prime plant immune responses [152,153]. These technologies are being designed with built-in evolutionary safeguards, such as targeting conserved pathogen genes that are less likely to mutate without fitness costs.

Precision agriculture tools are enabling unprecedented levels of disease monitoring and intervention. Advanced spectral imaging systems in research settings can detect pre-symptomatic infections with 85% accuracy as early as 5 days post-inoculation [154,155], showing the potential for timely interventions. IoT-based microclimate monitoring networks provide real-time data on temperature, humidity, and leaf wetness at the canopy level [156,157], improving disease prediction models. Next-generation predictive systems integrate pathogen genomic data with meteorological information and machine learning algorithms to forecast disease outbreaks at field-scale resolution. These systems are being coupled with automated application technologies that treat only high-risk zones to reduce chemical use.

Novel formulations are overcoming traditional limitations of disease control products. Nanocarrier systems using chitosan nanoparticles improve fungicide adhesion and rain fastness while enabling controlled release over 10–14 days [158,159]. RNAi-based biopesticides targeting *P. infestans* cellulose synthase genes have shown high efficacy in recent trials [32,151], with formulations designed to protect the fragile RNA molecules from environmental degradation. Smart delivery systems that activate only under specific pH conditions or in response to pathogen enzymes are being developed to maximize target specificity and minimize environmental impact. These include “stealth” formulations that remain inert until encountering infection sites. The key characteristics of these strategies are summarized for comparison in Table 3.

**Table 3.** A systematic comparison of the primary strategies available for managing potato late blight, ranging from conventional approaches to emerging technologies. Each strategy is evaluated based on its core mechanism of action, key advantages for disease control and sustainability, and major practical or biological limitations. The comparison highlights critical trade-offs, such as the durability of host resistance versus the rapid efficacy of fungicides, and the sustainability of ecological methods versus their variable performance.

Strategy	Core Mechanism	Key Advantages	Major Limitations & Practical Barriers
**Host Resistance** (6.1)	Deployment of R genes and QTLs to recognize pathogen or limit infection.	Reduces/eliminates fungicide need; cost-effective long-term.	Rapid breakdown of R genes; higher seed cost; potential yield/quality trade-offs.
**Fungicide Applications** (6.2)	Direct chemical inhibition of pathogen growth and reproduction.	Rapid, highly effective control during epidemics; broad availability.	Risk of resistance development; environmental and residue concerns; recurring cost and need for precise application.
**Ecological Approaches** (6.3)	Harnessing agronomic practices (rotation, diversification) and biocontrol agents to suppress disease.	Enhanced sustainability; improves soil health; reduces selection pressure for resistance.	Requires more knowledge; efficacy can be variable and context-dependent; not standalone solutions under high pressure.
**Emerging Technologies** (6.4)	Genetic editing (CRISPR), RNAi, nanocarriers, and precision agriculture for targeted intervention.	High specificity; potential for durability; reduces chemical inputs.	High R&D costs; stringent regulatory hurdles; limited access for resource-poor farmers; public acceptance issues.

### Practical implementation barriers

While an extensive array of management strategies exists, their translation from concept to widespread field application faces significant practical barriers. Economic constraints are paramount, especially for smallholder farmers who produce most of the potatoes in developing countries. Improved resistant varieties can cost 3–5 times more than conventional seeds, and the recurring expense of effective fungicide programs is often prohibitive.

Infrastructural and regulatory limitations further hinder adoption. Regulatory systems often struggle to keep pace with innovation, delaying the deployment of emerging technologies like CRISPR-edited crops or RNAi-based biopesticides, with approval processes averaging 5–7 years. Similarly, implementing precision agriculture tools and advanced application technologies (e.g. UAVs, electrostatic sprayers) requires a level of technical infrastructure and capital investment not universally available.

Finally, critical hurdles exist in knowledge transfer and labor dynamics. Current extension systems frequently fail to disseminate modern management knowledge to most growers, while widespread agricultural labor shortages complicate the implementation of labor-intensive practices essential for effective scouting and timely intervention. Addressing these multifaceted barriers is as vital as developing new technologies for achieving sustainable and equitable late blight management.

## Wild relatives as genetic resources and the role of alternative hosts

Wild *Solanaceae* species play a dual role in the interaction of *P. infestans* with hosts, serving as both valuable sources of genetic resistance and potential reservoirs for pathogen evolution. The wild relatives of cultivated potato, particularly those originating from the potato’s center of origin in Andes [160,161] and pathogen’s center of diversity in the Toluca Valley of Mexico, have co-evolved with *P. infestans* and developed robust defense mechanisms [162,163]. These wild relatives employ sophisticated immune strategies, including pathogen recognition via unconventional receptors and defense priming upon detection of conserved microbial patterns. For example, species such as *S. demissum* and *S. bulbocastanum* possess major R genes (specifically (R1-R11 and RB/Rpi-blb1, respectively) immuning to *P. infestans*, while others like *S. microdontum* exhibit broad-spectrum resistance through enhanced physical barriers and chemical defenses [164,165]. Pan-genome analyses have also identified dozens of novel resistance gene candidates across > 20 wild species [166,167], offering new opportunities for durable resistance breeding.

However, the very biodiversity that provides these genetic resources also supports pathogen persistence and adaptation. Alternative hosts, particularly weedy nightshades and bittersweet, create “green bridges” that maintain *P. infestans* inoculum between potato cropping seasons. Field studies across Europe indicate that nightshades species are present in up to one-third of agricultural field margins [168], serving as year-round reservoirs for the pathogen. Perhaps more critically, these alternative hosts function as evolutionary testing grounds where *P. infestans* experiments with new virulence combinations. For example, *S. nigrum* populations have been shown to select for Avr2 effector variants that later emerge in potato fields while *S. arrachoides* facilitates recombination between mitochondrial haplotypes [169,170]. The ability of *P. infestans* to infect these related but ecologically distinct hosts contributes to its remarkable adaptability, with some nightshade-adapted strains exhibiting expanded thermal tolerance or host ranges. Systematic removal of alternative hosts like *S. dulcamara* within 500 m of production fields eliminates important green bridges for pathogen survival between seasons [171].

This complex interplay presents both opportunities and challenges for disease management. On one hand, wild species offer unparalleled genetic diversity for resistance breeding, as demonstrated by the successful deployment of *S. americanum*-derived *Rpi-amr1* [172], which recognizes a conserved CRN effector motif. Modern tools like CRISPR-Cas9 now enable precise introgression of these wild resistance genes while minimizing linkage drag. On the other hand, the epidemiological role of alternative hosts necessitates integrated management strategies that address pathogen evolution across entire landscapes. This may include sanitation programs to remove nightshades from production areas, regional monitoring of pathogen populations in wild hosts, and crop rotation schemes designed to disrupt host connectivity.

Moving forward, researchers must adopt a more holistic understanding of the *P. infestans* pathosystem that considers both the genetic potential of wild relatives and the ecological dynamics of alternative hosts. High-throughput phenotyping platforms can accelerate the identification of novel resistance traits in wild germplasm, while landscape genomic approaches may predict virulence trajectories as the pathogen moves between cultivated and wild hosts. Ultimately, sustainable late blight management will require balancing the utilization of wild genetic resources with strategies to minimize pathogen adaptation in alternative hosts, a challenge that demands collaboration between breeders, pathologists, and agroecologists.

## Challenges

Managing *P. infestans* is fraught with multifaceted challenges that complicate sustainable control. Climate change is altering disease dynamics, expanding the range of the pathogen and disrupting traditional forecasting models. Economic barriers such as the high cost of resistant cultivars and limited access to advanced technologies disproportionately affect smallholder farmers. Additionally, ecological trade-offs arise from intensive fungicide use and the dual role of wild *Solanum* species as genetic resources and pathogen reservoirs. Addressing these challenges requires holistic strategies that balance efficacy, equity, and environmental sustainability.

### Climate change complications

Global climate change is fundamentally transforming the dynamics of late blight epidemics. Temperature increases of 2–4°C are projected to expand the pathogen’s suitable habitat [168], particularly into higher latitude regions previously unaffected by severe outbreaks. Altered precipitation patterns are creating unexpected infection windows that disrupt traditional disease forecasting models. Elevated atmospheric CO_2_ levels may have dual effects, potentially enhancing pathogen aggressiveness while simultaneously reducing host plant defenses [171]. More frequent extreme weather events, including unseasonal rains followed by drought periods, are generating conditions that favor explosive disease development while complicating management timing. These climate-driven changes require complete reevaluation of existing control paradigms and the development of more adaptive management systems.

### Economic and implementation barriers

The translation of scientific advances into practical solutions faces substantial real-world obstacles. Smallholder farmers, who produce the majority of potatoes in developing countries, frequently lack access to improved resistant varieties, which typically cost 3–5 times more than conventional seeds [173]. Regulatory systems struggle to keep pace with innovation, with approval processes for biotechnological solutions taking an average 5–7 years, which are often longer than the timeframe for pathogen adaptation [174]. Current knowledge transfer systems fail to reach majority potato growers in developing regions [175], leaving them without access to modern management strategies. Additionally, widespread agricultural labor shortages in many countries complicate the implementation of intensive management practices required for effective late blight control, creating a pressing need for more labor-efficient solutions.

### Ecological trade-offs

Current management approaches often generate unintended ecological consequences that must be carefully considered. The widespread adoption of resistant cultivars, while effective in the short term, may reduce genetic diversity in farmer fields, potentially increasing vulnerability to other pests and diseases. Intensive fungicide use, particularly broad-spectrum chemistries, disrupts beneficial soil microbiomes and may lead to secondary pest outbreaks. Conservation efforts for wild *Solanum* species, although important genetic resources for future breeding, sometimes conflict with the need to eliminate alternative hosts that serve as pathogen reservoirs. Even biological control methods can create ecological imbalances, as introduced antagonistic organisms may affect non-target species in complex agroecosystems [176]. These trade-offs highlight the need for more holistic, systems-based approaches to late blight management that consider entire agricultural ecosystems rather than focusing solely on pathogen control.

## Key outstanding questions

Despite advances in understanding *P. infestans*, critical knowledge gaps persist. Key questions revolve around effector evolution, environmental plasticity, and the durability of emerging technologies. The pathogen’s ability to adapt to climate change and overcome resistance mechanisms demands deeper exploration. Socioeconomic barriers to implementing solutions also warrant attention, particularly for resource-limited farmers. Answering these questions will require interdisciplinary collaboration to develop resilient strategies against this ever-evolving threat.

### Effector evolution & host adaptation

Several critical questions remain about how *P. infestans* manages its effector evolving repertoire evolution. Researchers are particularly interested in understanding how the pathogen balances the need for effector diversification to evade host recognition while maintaining essential virulence functions. Are certain effector families like RXLRs more evolutionarily labile than CRNs or apoplastic effectors? Another key question focuses on whether wild *Solanum* species with unconventional immune receptors could uncover previously unknown effector targets that might be exploited for more durable engineered resistance. These wild relatives may hold the key to identify conserved pathogen vulnerabilities that could be targeted across multiple *Phytophthora* species.

### Environmental plasticity & climate change

The pathogen’s response to changing climate patterns presents pressing research questions. Will rising temperatures primarily select for strains with expanded thermal tolerance thresholds, or will shifts in humidity and precipitation patterns prove more influential in determining future disease distribution? Another crucial question is how increasingly common extreme weather events, particularly alternating cycles of drought and flooding, may disrupt the pathogen’s life cycle and alter epidemic dynamics. Understanding these climate-pathogen interactions is essential for developing predictive models and adaptive management strategies.

### Durability of control strategies

Emerging technologies raise important questions about long-term efficacy. Can CRISPR-edited crops targeting conserved effector domains provide more durable resistance than traditional R genes, and if so, what are the optimal targets? For RNAi-based control methods, significant questions remain about potential off-target effects in field environments, including impacts on non-target organisms and the risk of inducing unintended gene silencing in host plants.

### Ecological & evolutionary trade-offs

Key questions focus on potential weaknesses in the pathogen biology that could be exploited. Does sexual reproduction, which generates such rich genetic diversity, come with fitness costs that could be targeted? Another important research direction is to examine how the pathogen compensates for impaired effector functions and whether there are predictable patterns. Understanding these evolutionary trade-offs could reveal novel approaches to slow pathogen adaptation and inform smarter resistance gene stacking strategies.

### Socioeconomic barriers

Implementation challenges also raise critical research questions. Given current economic constraints, how can resource-limited farmers gain access to advanced diagnostics and resistant varieties? What types of policy interventions – from subsidy programs to regional cooperation frameworks – are most effective in accelerating adoption of integrated management approaches? These questions require research into both technological solutions and social systems that enable equitable access to disease control innovations.

Addressing these multifaceted questions demands unprecedented collaboration across disciplines. Genomicists must work with field ecologists to connect molecular evolution to real-world pathogen behavior, while social scientists need to engage with biotechnologists to ensure solutions are both effective and implementable. Only through such integrated approaches can we hope to develop sustainable strategies that keep pace with this notoriously adaptable pathogen. The answers to these questions will shape the next generation of late blight management systems, with implications for global food security in an era of climate change and agricultural transformation.

## Conclusion

*P. infestans* continues to pose one of the most complex challenges in plant pathology due to its extraordinary capacity to adapt and evolve against control measures. In this review, we systematically examine the intricate biology of this pathogen from its unique reproductive strategies to its sophisticated molecular mechanisms of infection. The success of the oomycete stems from its dynamic effector repertoire, including RXLR and CRN proteins that actively reprogram host physiology, combined with its ability to rapidly modify these virulence factors genetically and epigenetically. These properties, coupled with efficient aerial dispersal mechanisms, contribute its historical and ongoing impact on global potato and tomato production.

The challenges in managing late blight diseases have never been more complex. The genomic plasticity of this pathogen enables it to overcome both chemical controls and host resistance at an alarming rate, while climate change is altering traditional disease patterns [177,178] and expanding its geographical range [179]. Current strategies face limitations due to the rapid breakdown of resistance genes, development of fungicide resistance, and the environmental consequences of intensive chemical use. Moreover, the globalization of agricultural trade continues to facilitate the spread of aggressive strains across continents, often outpacing our ability to develop effective countermeasures. These issues are compounded by the need to balance disease control with sustainable farming practices and economic viability for producers [180].

Moving forward, a paradigm shift in late blight management is urgently needed. This requires integrating cutting-edge genomic tools with advanced agronomic practices and eco-evolutionary understanding [142,181]. We must invest in next-generation surveillance systems that combine molecular diagnostics with predictive modeling to anticipate pathogen evolution. Breeding programs should prioritize durable resistance strategies that go beyond single-gene approaches by incorporating gene pyramiding and quantitative resistance traits. Simultaneously, we need to accelerate the development of biological controls and precision application technologies [182,183] that reduce reliance on conventional fungicides. Crucially, these scientific advances must be coupled with knowledge-sharing networks that connect researchers, extension services, and growers worldwide. Only through such collaborative, multidisciplinary efforts formulated by evolutionary principles can we hope to establish sustainable management systems capable of withstanding the evolving threat of this relentless pathogen while ensuring global food security in a changing climate.

## Data Availability

Data sharing is not applicable to this article as no new data were created or analyzed in this study.=
